# Symptoms of common mental disorder and cognitive associations with seropositivity among a cohort of people coming for testing for HIV/AIDS in Goa, India: a cross-sectional survey

**DOI:** 10.1186/1471-2458-13-204

**Published:** 2013-03-07

**Authors:** Rosie Mayston, Vikram Patel, Melanie Abas, Priya Korgaonkar, Ramesh Paranjape, Savio Rodrigues, Martin Prince

**Affiliations:** 1 Health Service & Population Research Department, Institute of Psychiatry, Kings College London, London, UK; 2 Department of Nutrition and Public Health Intervention Research, London School of Hygiene & Tropical Medicine, London, UK; 3 Sangath, Goa, India; 4 National AIDS Research Institute, Pune, India; 5 Goa Medical College, Goa, India

## Abstract

**Background:**

The majority of research on HIV/AIDS and mental health has been carried out among clinical populations: the time of onset of comorbid depression and the mechanisms for this are therefore unclear. Although there is evidence to suggest that asymptomatic people living with HIV/AIDS exhibit some cognitive deficits, the prevalence of poor cognitive functioning among people in low income settings at an early, pre-clinical stage has not yet been investigated.

**Methods:**

We used a cross-sectional survey design to test the hypotheses that symptoms of Common Mental Disorder (CMD) and low scores on cognitive tests would be associated with seropositivity among participants coming for testing for HIV/AIDS. Participants were recruited at the time of coming for testing for HIV/AIDS; voluntary informed consent was sought for participation in research interviews and data linkage with HIV test results. Baseline questionnaires including sociodemographic variables and measures of mental health (PHQ-9, GAD-7, panic disorder questions, AUDIT and delayed word list learning and recall and animal naming test of verbal fluency) were administered by trained interviews. HIV status data was extracted from clinical records.

**Results:**

CMD and scoring below the educational norm on the test of verbal fluency were associated with testing positive for HIV/AIDS in bivariate analysis (OR = 2.26, 1.31-3.93; OR = 1.77, 1.26-2.48, respectively). After controlling for the effects of confounders, the association between CMD and seropositivity was no longer statistically significant (AOR = 1.56, 0.86-2.85). After adjusting for the effects of confounders, the association between low scores on the test of verbal fluency and seropositivity was retained (AOR = 1.77, 1.27-2.48).

**Conclusions:**

Our findings provide tentative evidence to suggest that low cognitive test scores (and possibly depressive symptoms) may be associated with HIV status among people who have yet to receive their HIV test results. Impaired cognitive functioning and depression-like symptoms may be the result of the same underlying neurological damage. CMD and cognitive impairment may overlap to a greater extent than previously assumed. If replicated, this may have implications for the way in which we measure and treat CMD and cognitive functioning among people living with HIV/AIDS.

## Background

High levels of common mental disorder have been identified among people living with HIV/AIDS in communities around the world [1-5], including in India [6]. There is evidence from low and middle income and high income settings that Common Mental Disorders (CMD) such as depression have an adverse impact upon adherence [7-9], as well as additional negative effects upon HIV-related clinical outcomes such as disease progression and survival, that are independent of the effects of common mental disorder upon adherence [10,11]. Neurocognitive impairment usually thought to be due to HIV-related damage to the frontostriatal region of the brain is generally found to occur among 20–37 percent of people living with HIV/AIDS [12] and is associated with HIV-associated dementia, disease severity [13], mortality [14], adherence to antiretroviral therapy [15] and employment [16]. The precise mechanisms for the high prevalence of comorbid neurocognitive impairment with HIV/AIDS are not well understood but recent studies have found reduced subcortical volumes and metabolite abnormalities among HIV-positive participants on cART [17].

Although diagnosis and life events related to diagnosis and disclosure undoubtedly contribute to the poor mental health of people living with HIV/AIDS [18,19], there is evidence to suggest that the presence of HIV in the brain may also play a role in producing depression-like symptoms [20]. The majority of research on HIV/AIDS and mental health has been carried out among clinical populations: the time of onset of comorbid depression and the mechanisms for this are therefore unclear. Although there is evidence to suggest that asymptomatic people living with HIV/AIDS exhibit some cognitive deficits [21], the prevalence of poor cognitive functioning among people in low income settings at an early, pre-clinical stage has not yet been investigated.

The few researchers who have measured depression among people coming for testing in low income settings have generally found a high prevalence (45 percent in an Indian sample [6]; 41 percent among pregnant women in South Africa [22]). Depression was not found to be associated with testing positive for HIV/AIDS in either these samples.

The limited data available on HIV-associated cognitive impairment in low income settings generally indicates lower cognitive functioning among seropositive participants as compared to seronegative controls [12]. Prevalence estimates of impairment vary- a multicentre WHO survey found impairment among symptomatic individuals of between 13 and 19 percent [21], whereas recent studies carried out in India, China, Uganda and Botswana comparing HIV-positive with HIV-negative controls reported prevalence of between 31 and 56 percent [12,23].

The aim of the Umeed study was to investigate the relationship between CMD symptoms cognitive functioning and HIV/AIDS among a cohort of people attending a public clinic for HIV-testing. At the time of interview, study participants were not aware of their HIV status. Given the evidence to support a biological pathway between HIV infection and common mental disorder and cognitive impairment, we hypothesised that CMD symptoms and cognitive impairment would predict testing positive for HIV/AIDS among a sample that were not yet aware of the outcome of their test.

## Methods

### Design

Analysis of cross-sectional data from the “Umeed” cohort is presented here. Participants were recruited at the time of attending for pre-test counselling and testing for HIV/AIDS at Goa Medical College. Structured interviews including questions about demographics, HIV-related factors, as well as measures of CMD symptoms and alcohol and substance misuse were carried out in the clinic setting at the time of attendance for pre-test counselling and testing. Participants were then followed up via routine records in order to obtain HIV status and attendance of post-test counselling data.

In order to test the hypothesis that CMD symptoms and/or cognitive impairment would predict seropositivity among those without knowledge of their HIV status, study participants who reported having previously been tested for HIV/AIDS and who had received a seropositive result were excluded from analyses (n = 57) presented in Tables 1, 2, 3, 4 and 5.

**Table 1:** Characteristics of the sample

	**Prevalence n (%)**	
Total	1874	
Male	880	(47.0)
Female	994	(53.0)
Age		
18–25	441	(23.5)
26–30	357	(19.1)
31–34	292	(15.6)
35–40	282	(15.0)
41–45	162	(8.6)
46–50	150	(18.0)
51+	190	(10.1)
Language		
Hindi	628	(33.5)
Konkani	1178	(62.9)
English	68	(3.6)
Religion		
Hindu	1370	(73.1)
Christian	295	(15.7)
Muslim	206	(11.0)
Other	2	(0.1)
State of origin		
Goa	1053	(56.1)
Other than Goa	821	(43.8)
Marital status		
Married/co-habiting	1315	(70.2)
Widowed	13	(0.7)
Separated/divorced	127	(6.8)
Never married	419	(22.3)
Education		
None	335	(17.9)
Primary school	471	(25.1)
Secondary school	915	(48.8)
Further education	153	(8.2)
CMD symptoms	100	(5.3)
Symptoms of major depression	76	(4.05)
Symptoms of generalised anxiety	20	(1.1)
Symptoms of panic	30	(1.6)
Symptoms of major depression + scored more than 5 on GAD-7	51	(2.7)
Symptoms of generalised anxiety + scored more than 5 on PHQ-9	19	(1.0)
Symptoms of panic + scored more than 5 on PHQ-9	27	(1.4)
Abstinent/occasional alcohol use	1636	(87.2)
Harmful alcohol use	240	(12.8)
Cognitive functioning		
Delayed recall- Scored below educational norm	427	(22.8)
Verbal fluency- Scored below educational norm	480	(25.6)
Experienced domestic violence in the last 12m		
Yes	116	(6.2)
Perpetrated domestic violence in the last 12m		
Yes	81	(4.3)
Frequency of social outings, ie. visiting a friend/relative		
Often (at least 1/week)	94	(5.0)
Sometimes (at least 1/month)	157	(8.4)
Rarely (less than once 1/month)	922	(49.2)
Never	701	(37.4)
Frequency of having friends/relatives coming to visit		
Often (at least 1/week)	112	(6.0)
Sometimes (at least 1/month)	470	(25.0)
Rarely (less than once 1/month)	815	(43.5)
Never	477	(25.5)
Ever experienced hunger due to lack of money		
Yes	347	(18.5)
Accompanied to the ICTC		
No	695	(37.01)
Plan to disclose HIV/AIDS test results		
Yes	1489	(79.5)
No	340	(18.1)
Don’t know	45	(2.4)
Knowledge of HIV prevention		
Thorough	1433	(76.5)
Partial/poor	441	(23.5)
Knowledge of HIV transmission		
Thorough	576	(30.7)
Partial	698	(37.3)
Poor	600	(32.0)
Internalised stigma v. people living with HIV/AIDS		
None	675	(36.0)
Moderate	607	(32.4)
Strong	592	(31.6)
Anticipated stigma if tested positive for HIV/AIDS		
None	602	(32.1)
Moderate	511	(27.3)
Strong	761	(40.6)
Ever injected a non-prescription drug		
Yes	2	(0.1)
Ever had sex		
No	293	15.20
Male who had sex with a man during the last year		
Yes	14	0.75
Ever had sex with someone other than your regular partner in last 12m		
Yes	225	(12.0)
Ever received cash, gifts or favours in exchange for sex		
Yes	33	(1.8)
Ever had sex with someone who was a commercial sex-worker		
Yes	90	(4.8)
Has experienced pain/burning with urination		
Yes	364	(19.4)
Genital sores		
Yes	119	(6.4)
Genital warts		
Yes	61	(3.3)
Genital discharge		
Yes	327	(17.5)
Believes likely to test positive for HIV/AIDS		
Yes	35	(1.9)
Don’t know	478	(25.5)
Have a child or partner who is HIV-positive		
Don’t know	106	(5.7)
Yes	106	(5.7)
HIV status		
Positive	164	(8.8)

**Table 2:** Bivariate associations of with testing positive for HIV/AIDS

	**HIV-positive n (%)**		**OR (95% CI)**	**p**
Total	164	(8.75)		
*Demographics*				
Male	78	(8.86)	1.00	0.97
Female	86	(8.65)	0.97 (0.71–1.34)	
Age				
18–25	17	(3.85)	1.09 (1.00–1.18)	0.04
26–30	35	(9.80)		
31–34	32	(10.96)		
35–40	33	(11.70)		
41–45	18	(11.11)		
46–50	17	(11.33)		
51+	12	(6.32)		
Language				
Hindi	72	(11.46)	1.00	
Konkani	91	(7.72)	0.65 (0.47–0.90)	0.01
English	1	(1.47)	0.12 (.0.02–0.85)	0.01
State of origin				
Goa	69	(6.55)	1.00	<0.01
Other than Goa	95	(11.57)	1.87 (1.35–2.58)	
Marital status				
Married/co-habiting	119	(9.05)	1.00	
Widowed	23	(18.11)	2.22 (1.36–3.63)	<0.01
Separated/divorced	3	(23.08)	3.01 (0.82–11.13)	0.08
Never married	19	(4.53)	0.48 (0.29–0.79)	<0.01
Education				
None	46	(13.73)	0.70 (0.58–0.84)	<0.01
Primary school	44	(9.34)		
Secondary school	66	(7.21)		
Further education	8	(5.23)		
*Mental Health and Social Support*				
No/mild CMD symptoms	147	(8.29)	1.00	<0.01
Moderate/severe CMD symptoms	17	(17.00)	2.27 (1.31–3.93)	
Abstinent/occasional alcohol use	137	(8.38)	1.00	0.14
Harmful alcohol use	27	(11.25)	1.39 (0.89–2.14)	
Cognitive functioning			2.47 (1.40–4.34)	
Delayed recall- Scored above educational norm	117	(8.09)	1.00	
Delayed recall- Scored below educational norm	47	(11.01)	1.41 (0.98–2.01)	0.06
Verbal fluency- Scored above educational norm	104	(7.46)	1.00	
Verbal fluency- Scored below educational norm	60	(12.50)	1.77 (1.26–2.48)	<0.01
Frequency of social outings, ie. visiting a friend/relative				
Often (at least 1/week)	3	(3.19)	1.53 (1.25–1.88)	<0.01
Sometimes (at least 1/month)	14	(8.92)		
Rarely (less than once 1/month)	56	(6.07)		
Never	91	(12.98)		
Frequency of having friends/relatives coming to visit				
Often (at least 1/week)	8	(7.14)	1.38 (1.14–1.66)	<0.01
Sometimes (at least 1/month)	34	(7.23)		
Rarely (less than once 1/month)	56	(6.87)		
Never	66	(13.84)		
Ever experienced hunger due to lack of money				
No	112	(7.33)	1.00	<0.01
Yes	52	(14.99)	2.23 (1.56–3.17)	
Plan to disclose HIV/AIDS test results				
Yes	116	(7.79)	1.00	
No	44	(12.94)	1.76 (1.22–2.55)	<0.01
Don’t know	4	(8.89)	1.15 (0.41–3.28)	0.79
Ever had sex				
No	12	(4.10)	1.00	<0.01
Yes	152	(9.61)	2.49 (1.36–4.55)	<0.01
Ever had sex with someone other than your regular partner in last 12m				
No	136	(8.25)	1.00	0.04
Yes	28	(12.44)	1.58 (1.02–2.44)	
Ever had sex in exchange for money, favours or gifts				
No	157	(8.53)	1.00	0.01
Yes	7	(21.21)	2.89 (1.23–6.77)	
Ever had genital warts				
No	153	(8.44)	1.00	0.01
Yes	11	(18.03)	2.39 (1.22–4.69)	
Believes likely to test positive for HIV/AIDS				
No	72	(5.29)	1.00	<0.01
Don’t know	78	(16.32)	3.49 (2.47–4.93)	
Yes	14	(40.00)	11.94 (5.72–24.91)	
Have a child or partner who is HIV-positive				
No	103	(6.20)	1.00	
Don’t know	18	(16.82)	3.06 (1.77–5.29)	<0.01
Yes	43	(40.57)	10.32 (6.54–16.30)	<0.01

**Table 3:** Multivariate models adjusting for confounders of association between CMD symptoms and testing seropositive

**Model**	**Description**	**Adjusted OR (95% confidence interval)**	**P-value**
1	CMD symptoms	2.27 (1.31-3.92)	<0.01
2	Model 1 + education	2.04 (1.18-3.56)	0.01
3	Model 2 + experience of hunger due to lack of money	1.84 (1.05-3.22)	0.03
4	Model 3 + disclosure plans	1.73 (0.99-3.05)	0.06
5	Model 4 + genital warts	1.65 (0.93-2.92)	0.09
6	Model 5 + perceived likelihood of testing positive	1.50 (0.83-2.73)	0.18
7	Model 6 + cognitive functioning: verbal fluency	1.56 (0.86-2.85)	0.14

**Table 4:** Multivariate models adjusting for confounders of association between low cognitive functioning score (test 1) and testing seropositive

**Model**	**Description**	**Adjusted OR (95% confidence interval)**	**P-value**
1	Cognitive functioning: test 1	1.41 (0.98-2.01)	0.06
2	Model 1 + age	1.37 (0.96-1.97)	0.08
3	Model 2 + education	1.33 (0.93-1.90)	0.12
4	Model 3 + experience of hunger due to lack of money	1.26 (0.88-1.82)	0.21
5	Model 4 + perceived likelihood of testing positive	1.17 (0.80-1.70)	0.42
6	Model 5 + cognitive functioning test: verbal fluency	1.02 (0.68-1.51)	0.94

**Table 5:** Multivariate models adjusting for confounders of association between low cognitive functioning score : verbal fluency and testing seropositive

**Model**	**Description**	**Adjusted OR (95% confidence interval)**	**P-value**
1	Cognitive functioning: verbal fluency	1.77 (1.27-2.48)	<0.01
2	Model 1 + age	1.76 (1.26-2.47)	<0.01
3	Model 2 + education	1.73 (1.23-2.42)	<0.02
4	Model 3 + language	1.65 (1.18-2.33)	<0.01
5	Model 4 + marital status	1.71 (1.21-2.40)	<0.01
6	Model 5 + CMD symptoms	1.74 (1.24-2.46)	<0.01
7	Model 6 + has child/partner who is living with HIV/AIDS	1.63 (1.13-2.34)	0.01
8	Model 7 + perceived likelihood of testing positive	1.61 (1.12-2.33)	0.01

### Setting

Goa, the smallest Indian state by area, is situated on the west coast between Maharashtra and Karnataka and has a population of 1.34 millions (Government of Goa, 2010). For HIV/AIDS surveillance, Goa is divided into two districts: the north is described as one of India’s high prevalence districts, with more than one percent prevalence among women at antenatal care. The south is medium prevalence, with more than five percent prevalence found among those attending STI clinics (high risk group) (UNAIDS, 2008).

### Recruitment and measures

Participants were recruited at the time of attending the largest public Integrated Counselling & Testing Centre (ICTC) for pre-test counselling and testing. In 2008, 20.8 percent of attendees were “walk-in”, self-referrals, whilst the majority of people tested attended upon the recommendation of a Doctor (Goa State AIDS Control Society, 2012). Doctors refer participants for testing for a variety of reasons, including: unexplained or suspected HIV-related symptoms, pre-operative tests. Self-referrals include those who believe themselves to be at high-risk as well as those undergoing pre-marital tests or testing before going to work abroad. Potential participants were informed about the study by trained research assistants; consent for participation and data linkage with test results was sought. The study was approved by local and international research ethics committees (Sangath and KCL).

Questionnaires, administered by research assistants using palmtop computers were carried out at the time of participants coming for testing. In order to maximise privacy and quietness, interviews were carried out in purpose-built temporary cabins situated in the clinic reception area. People attending the ICTC for pre-test counselling and testing for HIV/AIDS were eligible to participate if they met the following inclusion criteria: demonstration of capacity- understanding the aims of the study and demands of participation and able to give informed consent; fluent in one of three local languages (Konkani, Hindi & English); aged more than eighteen years of age. Questions relating to demographics, HIV-related behaviours, beliefs and knowledge (transmission and prevention knowledge, disclosure plans, sexual behaviour, knowledge and symptoms of sexually transmitted infections) and measures of mental health were included in baseline interviews.

In order to access free-of-charge antiretroviral treatment services, it is necessary to present a test certificate from a state-run testing centre. Therefore, testing in the private sector, followed by re-testing at a public testing centre is relatively common. Participants were therefore asked if they had previously undertaken a HIV-test; those who had received test results were asked to disclose the result to interviewers.

### CMD and alcohol use

A modified version of the Patient Health Questionnaire, the brief PHQ-9, in combination with the 7-item Generalised Anxiety Disorder scale (GAD-7), plus the panic disorder module [24] were included in the baseline questionnaire to measure symptoms of depression, anxiety and panic. In a study comparing the sensitivity and specificity of five screeners for CMD in primary care which carried out in Goa, the PHQ was one of five instruments to achieve an AUC from ROC analysis of at least 0.80 (PHQ AUC = 0.84) [25]. In Umeed, as recommended by Spitzer et al. [24], a cut-off of 10 was used as an indicator for symptoms of major depression. A cut-off of 10 was used to indicate symptoms of severe generalised anxiety [26]. Participants reporting “yes” to each of the first four questions relating to anxiety attacks were indicated as panic disorder cases in the Umeed study. At the time of conducting the study, there were no measures of anxiety symptoms that had been validated in a local HIV-affected population. Therefore, we decided to use brief measures that showed good validity in primary care populations elsewhere. Given the evidence to support distinctive diagnostic categories of depression and anxiety [27-29], participants identified as having symptoms of major depression and/or generalised anxiety disorder and/or panic disorder were categorised as having common mental disorder during analysis. “CMD symptoms” does not refer to a clinical diagnosis but to the result of the screening procedure used in this epidemiological study.

The AUDIT is a 10 item questionnaire designed to measure medium (cut-off of eight) and high levels (cut-off of 16) of alcohol problems (WHO 2001). The instrument has been widely used in India ie. [30,31] and in studies conducted in Goa [32,33]. A recent validity study conducted in Goa demonstrated good sensitivity and specificity for the AUDIT, with an area under the curve of more than 0.80 [34]. For analysis, participants were categorised as hazardous drinkers if they scored above eight.

### Cognitive functioning

Two measures of cognitive functioning were included in the Umeed questionnaire: word list learning (assessing memory) and animal naming (measuring verbal fluency, an aspect of executive functioning, known to be a common deficit in people living with HIV/AIDS- [35]). The terms “low cognitive functioning” and “cognitive deficits” apply to the two domains tested only. Due to the broader aims of Umeed (investigating impact of psychological factors upon access to care) and the practicalities of measuring cognition in a busy clinic setting, we used brief, simple screeners to measure domains that may be impacted by HIV and that were most likely to be implicated in determining access to services.

For the word list learning test, interviewers read out a ten word list (adapted for use in India- [36]) three times. Participants were then asked to recite the list of words after each reading and finally requested to recall words after another section of the questionnaire had been completed. The final total recalled was recorded. For the animal naming test, participants were asked to tell the interviewer as many different types of animals as they could recall during one minute.

These tests originate from the Consortium to Establish a Registry of Alzheimer’s Disease (CERAD) test battery and have been validated among an older population in Goa as part of the validation of a cross-cultural diagnostic instrument for dementia [37]. Normative data was available, in the form of education-specific norms derived from the 10/66 study data for the youngest of the older age-group (60–64 years). Education-specific cut-points for possible cognitive impairment at 1.5 standard deviations below the norms were used in the Umeed study as indicators of low cognitive function.

### Outcome measures

Outcome data was collected from routine clinical records maintained by ICTC staff by an Umeed team member who was not involved in baseline data collection. HIV status and date of attendance of post-test counselling (if attended) was extracted from ICTC records. Study participants records were identified using the unique identifier assigned to participants by counsellors at pre-test counselling.

### Analysis

Bivariate analysis was carried out. chi-squared tests were used to test differences between distribution of HIV status outcome between categories of a) hypothesised exposures of interest (common mental disorder, cognitive functioning) and b) potential demographic, HIV-related and psychosocial confounders. Mantel Haenszel odds ratios comparing odds of testing positive for HIV among exposure categories found to be associated with seropositivity in chi-squared analyses are presented in Table 1 with 95 percent confidence intervals.

Where there was evidence from bivariate analysis of an association between exposures of interest and testing positive for HIV/AIDS, multivariate analysis was carried out to further test the hypotheses that CMD symptoms and cognitive impairment would predict seropositivity. Exposures of interest were entered singly into logistic regression models. Potential confounders were then entered one-by-one. Criteria for inclusion in final models were: a) variables considered a priori confounders (for example, association of age with cognitive functioning); or b) variables selected using a “change in estimate” method ie. those that resulted in a significant change in the effect of exposure of interest upon outcome (more than 10 percent change in odds ratio).

## Results

### Characteristics of the sample

As described in Table 1, 8.8 percent of the sample tested seropositive. The majority of the sample were female (53.0 percent); the mean age of participants was 35 years. Almost three quarters were Hindu (73.0 percent) and the majority were born in Goa (56.1 percent). Most were married (70.2 percent); 25.1 percent had attended up to primary school whilst 17.9 percent had received no education at all. 18.5 percent reported ever having experienced hunger due to lack of money.

The prevalence of CMD symptoms among this sample of people of people coming for testing for HIV/AIDS was 5.3 percent. 12.8 percent of participants reported hazardous alcohol use (26.4 percent among men- not presented). Around a quarter of the sample scored below the educational norm on two cognitive tests of delayed recall (22.8 percent) and verbal fluency (25.6 percent).

### Analysis of associations with testing positive for HIV/AIDS

As described in Table 2, in bivariate analysis, CMD symptoms and scoring below the educational norm on verbal fluency test were associated with testing positive (OR = 2.27, 1.31-3.93; OR = 1.77, 1.26-2.48, respectively). There was a trend towards an association between scoring below educational norm on the test of delayed recall and seropositivity (OR = 1.41, 0.98-2.01). Hazardous alcohol use was not associated with seropositivity.

All variables found to be associated with testing positive are presented in Table 2. Sociodemographic variables associated with testing positive included: age (OR = 1.09, 1.00-1.18), language (those speaking Konkani or English were less likely to test positive than those speaking Hindi: OR = 0.65, 0.47-0.90; OR = 0.12, 0.02-0.85, respectively), being born outside of Goa (OR = 1.87, 1.35-2.58), being widowed (OR = 2.22, 1.36-3.63), having experienced hunger due to lack of money (OR = 2.23, 1.56-3.63). Visiting relatives or friends (or receiving visits) less than once a week was associated with seropositivity (OR = 1.53, 1.25–1.88, OR = 1.38, 1.14-1.66, respectively). Education was associated with a reduced likelihood of testing positive (OR = 0.70, 0.58-0.84).

A number of HIV-related variables were also associated with seropositivity, including: planning not to disclose test results (OR = 1.76, 1.22-2.55), having sex with someone other than a regular partner (OR = 1.58, 1.02-2.44), having sex in exchange for cash, gifts or favours (OR = 2.89, 1.23-6.77), believing it is likely that the test result will be positive (OR = 11.94, 5.72-24.91), having a child/partner who is living with HIV/AIDS (OR = 10.32, 6.54-16.30).

Variables tested but not found to be associated with HIV status (results not presented in table) included: internalised (p = 0.16) and anticipated stigma (p = 0.40), knowledge of HIV prevention (0.38) and transmission (p = 0.18).

Table 3 shows that, after controlling for the effects of confounders (education, experience of hunger due to lack of money, disclosure plans, genital warts, perceived likelihood of testing positive and verbal fluency), the association between CMD symptoms and seropositivity is no longer statistically significant (AOR = 1.56, 0..86-2.85). As presented in Table 4, the trend towards an association between delayed recall and testing positive is eliminated by the addition of confounding variables (age, education, experience of hunger due to lack of money, perceived likelihood of testing positive, verbal fluency) to the model (AOR = 1.02, 0.68-1.51).

However, as can be seen in Table 5, although the addition of potential confounders of the association between verbal fluency and seropositivity (age, education, language, marital status, CMD symptoms, having a child/partner who is seropositive, perceived likelihood of testing positive) reduced the effect size, in the final model, verbal fluency remains significantly associated with testing positive for HIV/AIDS (AOR = 1.77, 1.27-2.48).

## Discussion

The Umeed cohort is the largest study to describe the mental health of people coming for testing for HIV/AIDS in a low income setting. The inclusion of seropositive and seronegative attendees of HIV testing provided us with a unique opportunity to test hypotheses about mental health and HIV status in the absence of the impact of diagnosis and consequent experiences of living with HIV/AIDS upon participant’s mental health. We hypothesised that CMD symptoms and cognitive impairment would be associated with testing positive. Our findings were inconclusive. Although CMD symptoms was strongly associated with testing positive in bivariate analyses, after adjustment for confounders, the effect size of this association was reduced and no longer statistically significant. We found some evidence to support the hypothesised association between low cognitive functioning and seropositivity. Although there was only a bivariate trend towards an association between delayed recall and seropositivity, scoring below education-adjusted norms on the test of verbal fluency (animal naming) was associated with testing positive for HIV/AIDS (AOR = 1.77, 1.27-2.48).

Almost all of the large body of research about HIV/AIDS and CMD in low and middle income settings has been carried out among single groups, with a HIV-positive diagnosis, without comparison with seronegative controls (for example, [5,38,39]). A few studies have compared prevalence of depression between seropositive and seronegative groups (ie. [4,40]). In one of the few studies similar in design to Umeed (a sample of test-seekers in Pune where 45.5 percent subsequently received a positive diagnosis), univariate analyses revealed no evidence of an association between common mental disorder and seropositivity [6]. In the Umeed sample, bivariate analysis revealed a strong association between CMD and testing HIV-positive (OR = 2.27, 1.31-3.92). However, after the gradual introduction of covariates into the model, the effect size was reduced and no longer statistically significant (AOR = 1.56, 0.86-2.85).

Although the multivariate analysis presented in Table 3 demonstrates that the association between CMD symptoms and seropositivity was partially confounded by sociodemographic and HIV-related covariates, we should be cautious before interpreting these findings as a demonstration that there was no true association between CMD symptoms and seropositivity. As indicated by the relatively wide confidence intervals presented in Table 3, the low prevalence of both CMD and seropositivity among Umeed participants (and particularly in the sub-sample used for this analysis) resulted in reduced power to detect an association, therefore we cannot rule out the existence of a true association between CMD and seropositivity.

As described, the prevalence of CMD symptoms identified among the Umeed sample was much lower than that reported among the two other samples of people coming for testing in low and middle income settings described in the literature. The relatively low prevalence of HIV/AIDS measured in Umeed as compared to these other samples (45.5% in India; 40.9% among pregnant women in South Africa) may help to explain the difference in prevalence of CMD symptoms. The Umeed sample is heterogeneous and includes participants at high and low-risk of testing positive. Therefore, it is plausible that the overall prevalence in the Umeed sample may be more similar to that found in the local community [41], rather than that identified in primary care [42]. Without further research among people coming for testing in similar settings, it is difficult to contextualise our findings further. Of course, the possibility that our mode of measurement of CMD symptoms may have contributed to the low reported prevalence of CMD symptoms cannot be ruled out. Although the PHQ-9 is commonly used in Western settings, the lack of validation of cut-points for use among HIV-affected populations in low income settings may have had an impact upon the low prevalence of common mental disorder identified in the Umeed cohort. As described, all interviews were carried out in temporary, private cabins. All participants were given detailed information about CMD symptoms and the aims and objectives of the study; the PHQ has been found to be valid among a Goan sample of people attending primary care. However, the possibility remains that using the PHQ-9 immediately prior to testing in the busy clinic setting may have inhibited participants willingness to disclose potentially sensitive information about symptoms of anxiety and depression. Our analysis demonstrated a high degree of co-morbidity of symptoms of anxiety and depression within our sample (of those scoring >10 on the GAD-7, 75 percent scored >10 on the PHQ-9; of those scoring >10 on the PHQ-9, 20 percent scored >10 on the GAD-7 and 47.4 percent scored between 5 and 10 on the GAD-7), thus supporting the dichotomous “symptoms of CMD” variable used in our analysis.

Given that HIV/AIDS is associated with morbidity, stigma and discrimination [43,44], poverty [45] and mortality [46,47], it is reasonable to expect that living with the disease contributes greatly to the prevalence of CMD among people with HIV/AIDS. However, there is evidence from neurological research to suggest that the inflammatory response that contributes to the neuronal loss that causes HIV-associated cognitive impairment may also trigger cytokine-induced depression [20]. Recent research suggests that even in the absence of other symptoms of depression and cognitive impairment, HIV-related damage to white matter may be associated with symptoms of apathy (“reduced self-initiated cognitive, emotional and behavioural activity”) [48]. In addition to social and environmental risk factors associated with their disease status, people living with HIV/AIDS (and other chronic conditions) may have a biological vulnerability to the development of depressive symptoms [46]. Although evidence suggesting that CMD may be associated with risky behaviour [47,49] means that a reverse relationship (pre-existing depression promoting sexual risk-taking and acquisition of HIV) cannot be ruled out, presence of genital warts and perception of HIV-risk were the only sexual risk-related variables, that confounded the association between CMD symptoms and seropositivity- therefore this pathway seems unlikely.

The Umeed finding that low scores on the test of verbal fluency were associated with seropositivity corresponds with the results of a recent meta-analysis that revealed a statistically significant deficit in mean scores on tests of verbal fluency among people living with HIV/AIDS, as compared to HIV-negative controls (−0.31, −0.44 to −0.18) [50]. In the only study to compare verbal fluency between seropositive individuals and seronegative controls in India, although HIV-positive individuals differed in other language-related domains (phonemic fluency, verbal working memory and verbal learning and memory); differences in animal naming fluency were not statistically significant [51]. Similarly, statistically significant deficits in verbal learning and memory were identified between seropositive individuals and seronegative controls in a previous south Indian study [52].

Neurological evidence supports the presence of cognitive deficits early in the course of the disease [12]. Both cognitive domains measured in Umeed (category fluency and verbal learning and memory) are consistent with diffuse or frontostriatal pathology, both of which may be characteristic of the effects of HIV-1 on the brain [50]. There is now a growing body of evidence on the cognitive profile of people living with HIV/AIDS in low income settings, including India [52]. Whereas small studies with detailed neuropsychological assessments tend to have stringent exclusion criteria which may limit the generalisability of findings, in Umeed, exclusion criteria were limited, meaning that our sample was likely to be more representative of the base population. The aim of Umeed was to measure cognitive functioning in the selected domains most likely to have an impact upon linkage to care and adherence and to examine whether these were associated with testing positive. The animal naming test of category fluency and the word list learning test of verbal learning and memory were selected as appropriate measures that would be relatively quick and simple for trained research assistants to administer among a large cohort of participants in a busy clinic setting.

The Umeed study design had several other methodological strengths. Unlike other studies that have investigated mental health and HIV/AIDS in a low income setting, the Umeed study was carried out among a large sample that included both seropositive and seronegative individuals recruited prior to diagnosis. Thus we were able to test hypotheses that CMD symptoms, cognitive functioning would be associated with seropositivity independently of the effects of diagnosis. Blind recruitment of seropositive and seronegative participants from the same clinic helped to ensure comparability of the two groups and reduced risk of selection bias. There was evidence from previous research to support the validity of measures of CMD and cognitive functioning among Goan populations.

However, there were also limitations. Due to ethical considerations, those who were potentially most unwell: (ie. unable to participate in 30 minute interview without experiencing undue distress or discomfort or lacking capacity to understand aims, participation demands and give informed, voluntary consent) were excluded from our sample (n = 102). Therefore, estimates for CMD symptoms, low cognitive functioning and seropositivity are likely to be lower than the true prevalence among people coming for testing for HIV/AIDS in Goa Medical College. Subsequently, this may have contributed to smaller effect sizes for associations between mental health exposures and HIV status.

It is possible that social desirability bias may have had an impact upon our measures of risk-taking behaviours. Reported risk-taking behaviours were fairly low relative to prevalence of HIV: 0.1% reported injecting drugs, 0.8% of men reported having had sex with other men, 1.9% reported having received money or gifts in exchange for sex; 5% reported having sex with a sex worker and 12.6% reported having sex with someone other than a regular partner in the last year. This may have limited our ability to control for the confounding effects of risk-taking behaviours upon observed associations between common mental disorder/low cognitive functioning and HIV status.

There were other limitations to our approach to cognitive testing. The cognitive screening instruments used in Umeed were chosen because they measured the domains most likely to have an impact upon access to care. The education-adjusted norms for cognitive functioning have not been validated among the younger Goan population or in the context of HIV/AIDS. We were unable to discriminate between sub-cortical deficits commonly caused by HIV and cognitive deficits with other causes (educational deprivation, mental sub-normality, language issues etc.), Unmeasured confounding is a possibility. Reverse causality cannot be ruled out. Low cognitive test scores may have been caused by a factor other than HIV that was associated with HIV-risk and acquisition. There is evidence to support a link between heavy alcohol use and poor cognitive functioning [53], as well as an association between alcohol-related impairment and reduced perception of sexual-risk [54]. However, given that we found little evidence of any association between alcohol use and HIV status, it seems unlikely that this pathway explains the observed relationship between low cognitive functioning and testing positive for HIV/AIDS.

Lack of detection of an association between delayed recall and seropositivity may be due to error in the measurement of delayed recall in Umeed. Although every effort was made to carry out interviews in an environment conducive to cognitive testing, it is possible that noise and interruptions to interviews had an impact, particularly on measurement of delayed recall, the more complex of the two cognitive tests. Measurement error may have led to some random misclassification of delayed recall test results, which may have contributed to masking a true association between delayed recall test results and testing positive.

## Conclusion

Umeed study findings provide tentative evidence that prior to knowledge of HIV status, low cognitive test scores (and perhaps CMD), may be associated with seropositivity among people coming for HIV-testing. Our findings suggest that a biological mechanism may contribute to the high prevalence of CMD and poor cognitive functioning commonly found among people living with HIV/AIDS. Impaired cognitive functioning and depression-like symptoms may be the result of the same underlying neurological damage caused by the presence of HIV in the brain. CMD and cognitive impairment may overlap to a greater extent than previously assumed. In order to clarify the relationship between CMD, cognitive impairment and HIV/AIDS, further research among pre-diagnosis samples is necessary. It would be helpful to include detailed assessments of key cognitive domains. If a closer interrelationship between cognitive functioning and CMD among people living with HIV/AIDS is demonstrated, this may have implications for the way in which we measure and treat CMD and cognitive functioning among people living with HIV/AIDS. It will be important to explore the possibility of early behavioural markers (such as apathy) as predictors of later cognitive decline. Depending on the outcome of future research, routine integration of screening for both CMD and cognitive deficits within Voluntary Counselling and Testing should be considered.

## Competing interests

The authors declare that they have no competing interests.

## Authors’ contributions

MP & RM conceived of the study and planned analyses. RM carried out analyses. VP & MA participated in the design of the study. RM & PK co-ordinated study staff and data collection. SR provided provided on-site supervision during data collection. RP advised on study design and ethical considerations. All authors read and approved the final manuscript.

## Pre-publication history

The pre-publication history for this paper can be accessed here:

http://www.biomedcentral.com/1471-2458/13/204/prepub
